# Translocation and fate of nanospheres in pheochromocytoma cells following exposure to synchrotron-sourced terahertz radiation

**DOI:** 10.1107/S1600577523004228

**Published:** 2023-06-20

**Authors:** Palalle G. Tharushi Perera, Zoltan Vilagosh, Denver Linklater, The Hong Phong Nguyen, Dominique Appadoo, Jitraporn Vongsvivut, Mark Tobin, Chaitali Dekiwadia, Rodney Croft, Elena P. Ivanova

**Affiliations:** a School of Science, RMIT University, GPO Box 2476, Melbourne, Victoria 3001, Australia; b School of Science, Computing and Engineering, Swinburne University of Technology, Melbourne, Victoria 3122, Australia; c Biomedical Engineering, Faculty of Engineering and Information Technology, University of Melbourne, Melbourne, Victoria 3010, Australia; d THz Beamline, Australian Synchrotron, 800 Blackburn Road, Melbourne, Victoria 3168, Australia; e IR Microspectroscopy, Australian Synchrotron, 800 Blackburn Road, Melbourne, Victoria 3168, Australia; f RMIT Microscopy and Microanalysis Facility, STEM College, RMIT University, Melbourne, Victoria 3000, Australia; g School of Psychology, Illawara and Medical Research Institute, University of Wollongong, Wollongong, New South Wales 2522, Australia; ESRF – The European Synchrotron, France

**Keywords:** fate of nanospheres, electromagnetic fields (EMFs), synchrotron-sourced THz radiation, PC 12 neuronal cells, membrane permeability

## Abstract

The routes by which foreign objects enter cells is well studied; however, their fate following uptake has not been explored extensively. Following exposure to synchrotron-sourced (SS) terahertz (THz) radiation, reversible membrane permeability has been demonstrated in eukaryotic cells by the uptake of nanospheres; nonetheless, cellular localization of the nanospheres remained unclear. This study utilized silica core-shell gold nanospheres (AuSi NS) of diameter 50 ± 5 nm to investigate the fate of nanospheres inside pheochromocytoma (PC 12) cells following SS THz exposure. Fluorescence microscopy was used to confirm nanosphere internalization following 10 min of SS THz exposure in the range 0.5–20 THz. Transmission electron microscopy followed by scanning transmission electron microscopy energy-dispersive spectroscopic (STEM-EDS) analysis was used to confirm the presence of AuSi NS in the cytoplasm or membrane, as single NS or in clusters (22% and 52%, respectively), with the remainder (26%) sequestered in vacuoles. Cellular uptake of NS in response to SS THz radiation could have suitable applications in a vast number of biomedical applications, regenerative medicine, vaccines, cancer therapy, gene and drug delivery.

## Abstract 2

Synchrotron-sourced radiation-driven internalization and localization of nanospheres inside mammalian cells.

## 1. Introduction

Terahertz technologies have gained attention in recent years given their prospects in a wide variety of applications ranging from telecommunications to material science (Gallerano & Biedron, 2004 ▸; Son *et al.*, 2019 ▸; Sitnikov *et al.*, 2018 ▸; Cocker *et al.*, 2021 ▸; Todorova *et al.*, 2016 ▸). The terahertz (THz) band lies between the microwave and infrared wavelengths on the electromagnetic spectrum (Todorova *et al.*, 2016 ▸), from 0.1 mm to 1 mm (1 THz = 10^12^ Hz) (Todorova *et al.*, 2016 ▸; Perera *et al.*, 2019 ▸). THz radiation can also be described by: wavelength (λ = 30–3000 µm); wavenumber (*k* = 3.3–334 cm^−1^); period (0.1–10 ps); temperature (*T* = 4.8–478 K); photon energy (*E* = 0.4–41 meV) (Wiatrak *et al.*, 2020 ▸). THz radiation differs from X-ray radiation as it is a non-ionizing radiation; THz frequencies do not carry enough energy to cause direct ionization effects on biological tissue (Wiatrak *et al.*, 2020 ▸). Ionization results in the formation of highly reactive free radicals that can cause indirect or secondary damage to biomolecules (Wiatrak *et al.*, 2020 ▸). Non-ionizing radiation, such as THz, does not generate free radicals but can cause thermal effects which cannot be distinguished from the biological effects that are caused by bulk heating (Wiatrak *et al.*, 2020 ▸). This presents a huge advantage over current techniques like X-ray based techniques which cause biomolecules to break down (Son *et al.*, 2019 ▸) thereby increasing its significance in potential clinical applications. Nanospheres (NS) of controlled geometry and surface chemical properties have proven to be of immense importance in theranostics. Their applications range from bioimaging of cells and tissues, immunoassays, clinical chemistry, targeted delivery of antigens, drugs and gene therapy (Gallerano & Biedron, 2004 ▸; Son *et al.*, 2019 ▸; Sitnikov *et al.*, 2018 ▸). New physico-chemical properties endowed by the nanoscale such as high surface-to-volume ratio is key to enabling (i) ease of functionalization, (ii) conjugation of biomolecules (Son *et al.*, 2019 ▸) and (iii) high X-ray absorption co-efficient (Cocker *et al.*, 2021 ▸). Gold (Au) NS have been rapidly adopted in diagnostic and therapeutic applications in recent years (Son *et al.*, 2019 ▸). For example, Au NS have been utilized in photodynamic therapy (PDT) because of properties such as effective fluorescence quenching combined with surface plasmon resonance (Sitnikov *et al.*, 2018 ▸). PDT is considered an effective treatment for oncological, skin and infectious diseases. Au NS have also been used as efficient sensors (Cocker *et al.*, 2021 ▸) for the detection of various molecules and metal ions (Sitnikov *et al.*, 2018 ▸).

The pheochromocytoma cell line PC 12 is routinely used in neuroscience research for studying neurotoxicity, neuro­inflammation and neurosecretion (Wiatrak *et al.*, 2020 ▸; Orlowska *et al.*, 2017 ▸; Jhawar *et al.*, 2022 ▸). In the presence of nerve growth factor, PC 12 cells can differentiate into sympathetic ganglion neurons on surfaces coated with various proteins for enhanced cellular attachment (Wiatrak *et al.*, 2020 ▸; Orlowska *et al.*, 2017 ▸; Jhawar *et al.*, 2022 ▸). While investigating the bioeffects of synchrotron-sourced (SS) THz radiation on PC 12 cells, it was found that SS THz induced cell membrane permeabilization (Perera *et al.*, 2019*a* ▸,*b* ▸), confirmed using fluorescence microscopy and transmission electron microscopy (TEM) of internalized fluorescent silica (Si) NS (Perera *et al.*, 2019*a* ▸). Notably, upon exposure to SS THz radiation, the intra-cellular physiology of the PC 12 cells remained unaffected (Perera *et al.*, 2019*a* ▸). Cellular assays were conducted to assess cell viability, metabolic status, protein concentration and neuronal differentiation for a period of seven days, with no detrimental effects observed except for membrane blebbing in PC 12 cells which was not evident in the untreated control sample (Perera *et al.*, 2019*a* ▸).

Microwave irradiation at a fixed frequency of 18 GHz has also been studied in eukaryotes, red blood cells (Nguyen *et al.*, 2017 ▸), model lipid membranes (Tharushi Perera *et al.*, 2021 ▸) and prokaryotes (Nguyen *et al.*, 2015 ▸; Shamis *et al.*, 2012*a* ▸,*b* ▸). Reversible membrane permeability was reported in PC 12 cells (Perera *et al.*, 2018 ▸), confirmed via the localization of Si core-shell Au NS (Tharushi Perera *et al.*, 2022 ▸). It was found that the AuSi NS internalized as a result of 18 GHz high-frequency electromagnetic energy (HF EME) were localized within the cytoplasm, within vacuoles inside the cytoplasm or were membrane-associated (Tharushi Perera *et al.*, 2022 ▸), whereas no internalization of NS was detected in the non-exposed PC 12 cells.

The aim of the current study was to investigate the cell permeabilization in response to SS THz radiation by locating the presence of AuSi NS. For this purpose we employed TEM, scanning transmission electron microscopy energy-dispersive spectroscopic (STEM-EDS) and fluorescence microscopy. Transportation of foreign material across a biological barrier, as well as understanding of the mechanism of cellular uptake leading to the intracellular route, are of utmost importance in the field of nanomedicine, drug and gene therapy (Florczak *et al.*, 2019 ▸). Accordingly, exploring the SS THz induced internalization and localization of NS in mammalian cells may increase the possibility of non-ionizing radiation becoming a remarkable tool in cell permeabilization techniques due to the lack of detrimental intracellular effects following exposure.

## 2. Materials and methods

### 2.1. PC 12 cell culture and growth conditions

Pheochromocytoma cells are derived from the rat adrenal medulla (Tharushi Perera *et al.*, 2022 ▸). The PC 12 cell line used in this study was purchased from the American Type Culture Collection (ATCC, USA) and cultured in a complete Gibco RPMI medium (Thermo Fisher Scientific, Australia) supplemented with 10% Gibco horse serum (Thermo Fisher Scientific, Australia, HS), 5% Gibco foetal bovine serum (Thermo Fisher Scientific, Australia, FBS) and 1% Gibco penicillin/streptomycin (Thermo Fisher Scientific, Australia). Supplements were stored as aliquots at −20°C. Stock solutions of the PC 12 cells were prepared in a medium containing 90% FBS and 10% DMSO and stored in liquid nitro­gen. The cells were maintained at 37°C with 5% CO_2_ in a 95% humidified incubator. The medium was changed every two days and passaged accordingly when the confluence reached 90%.

### 2.2. SS THz exposure at the beam extraction port

PC 12 cells were exposed to 10 min of SS THz radiation using the beam extraction port (BEP), located at the Australian Synchrotron (Melbourne, Victoria, Australia). A monolayer of PC 12 cells was constructed at a concentration of 7.7 × 10^6^ cells ml^−1^ using 30 µL in phosphate buffered saline (PBS) on a polyethyl­ene (PE) film using an O-ring sealed using grease.

**Figure 1:**
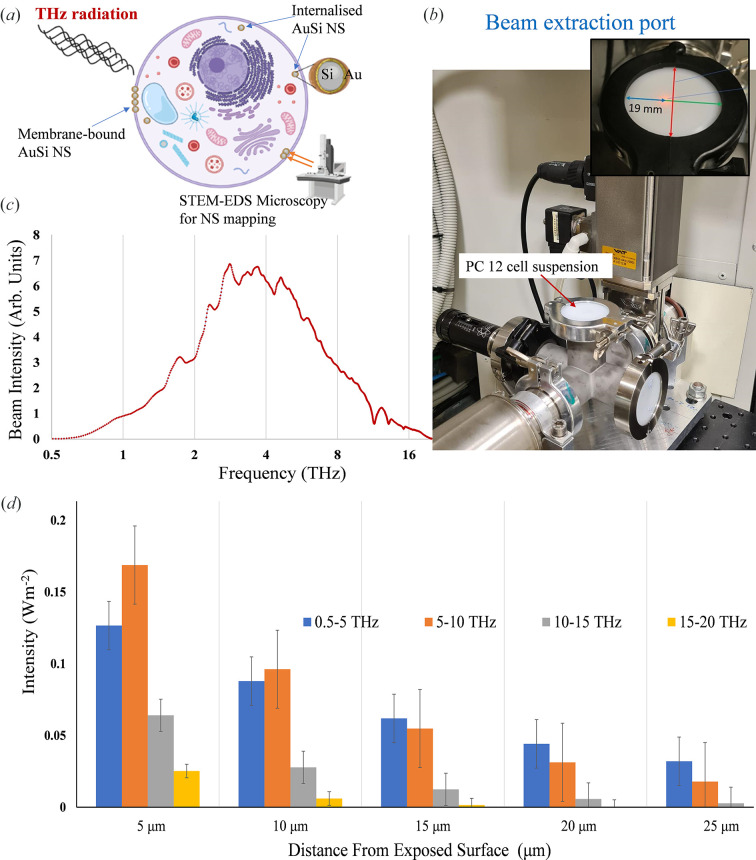
Overview of the experimental design and the technical setup at the Australian Synchrotron Far-IR/THz beamline. (*a*) Schematic representation of AuSi NS localization in PC 12. The SS THz exposed PC 12 samples were analysed for AuSi NPs following a 10 min exposure. (*b*) BEP at the Australian Synchrotron Far-IR/THz beamline, with the inset showing the beam axis in red; the beam is present within a 19 mm diameter (blue arrow) from the inner edge to the beam axis. The PC 12 cell suspension was placed on a polyethyl­ene window (red arrow) using an O-ring in the presence of the beam. (*c*) The spectrum of the intensity of the Australian Synchrotron THz beamline as shown by the resident Si bolometer; intensity is presented in arbitrary units, with a logarithmic scale for the THz frequencies (Vilagosh *et al.*, 2022 ▸). (*d*) Depth-dependent absorption pattern of the Australian Synchrotron THz beam. The two variables share an exponential relationship where the absorption of the beam decreases as the depth of the sample increases.

The present configuration of the Australian Synchrotron THz beam delivers energies approaching 0.058 mW over a 4.5 mm radius spot size giving a total intensity of ∼1.25 W m^−2^ (0.125 mW cm^−2^) at the BEP. After adjustments for the losses due to the sample holder, the intensity reduces to 1.0 W m^−2^. The Australian Synchrotron beam contains frequencies from 0.5 THz to above 20.0 THz with a variable intensity [Fig. 1 ▸(*c*)]. The spectrum of the intensity beam is centred on 4.0 THz with the half maximum points occurring at 2.0 THz and 8.0 THz. The temperature of the sample being exposed was recorded repeatedly using an IR gun (Fluke, Infrared thermometers, Everett, WA, USA) before and while the sample was being exposed. The temperature was recorded every minute for 10 min. After the exposure, the sample was collected from the PE film and analysed. Three repeats were carried out for post-exposure analysis. Furthermore, few independent technical replicates were performed over the course of time.

### 2.3. Internalization of silica nanospheres in PC 12 cells

Fluorescent silica nanospheres with a diameter of 23.5 ± 0.2 nm (FITC) (Corpuscular, Cold Spring, NY, USA) were used to study the permeability of PC 12 cells. The membrane phospho­lipids were stained using carbocyanine DIL (1,1′-diocta­decyl-3,3,3′,3′-tetra­methyl­indocarbocyanine perchlorate) dye (ThermoFisher Scientific, Australia). Immediately following SS THz exposure, the nanospheres were added to the cell suspension at a concentration of 10 µg ml^−1^. After 5 min of incubation, the samples were washed twice using PBS and centrifuged at 1300 rpm for 5 min at 25°C. The procedure was repeated for the untreated controls, where the cell samples were mixed with 10 µL of FITC nanosphere solution. A 150 µL aliquot of the sample was visualized using a Flouview FV10i-W inverted microscope (Olympus, Tokyo, Japan). Ten different fields of view were analysed per sample. Confocal laser scanning microscopy (CLSM) images were used in quantifying nanosphere uptake by counting the number of cells emitting the green fluorescence in parallel with the cells that are not permeabilized.

### 2.4. Localization of silica shelled gold nanospheres

Silica core-shell gold nanospheres with a diameter of 50 nm ± 5 nm (nanoComposix, San Diego, CA, USA) were used to study the uptake and localization of PC 12 cells. Immediately following SS THz exposure, the nanospheres were added to the cell suspension at a concentration of 10 µg ml^−1^. After 5 min of incubation, the samples were washed twice using PBS and centrifuged at 1300 rpm for 5 min at 25°C. The procedure was repeated for the untreated controls, where the cell samples were mixed with 10 µL of AuSi NS.

### 2.5. Sample preparation for TEM

After 5 min of incubation in the presence of nanospheres following SS THz exposure, cell suspensions were pelleted by centrifugation at 1300 rpm for 5 min at 25°C. The cells were then washed twice with PBS (10 m*M*, pH 7.4) to remove any unbound nanospheres. The cell pellet was conditioned with 0.1 *M* sodium cacodylate buffer (pH 7.4). The cell pellet was then re-suspended in primary fixative of 4% paraformaldehyde and 2.5% glutaraldehyde in 0.1 *M* sodium cacodylate buffer overnight at 4°C and washed thrice in cacodylate buffer for 10 min each. The cells were post-fixed in 1% osmium tetroxide (OSO_4_) and 1.5% potassium ferrocyanide for 1 h followed by three washes in distilled water for 10 min each. The cells were dehydrated using a graded series of ice-cold ethanol (50%, 70% and 90%) for 15 min each. The cells were further dehydrated by passing through 100% ethanol twice followed by 100% acetone twice for 30 min each. The cells were further infiltrated with a 1:1 ratio of acetone:Spurr’s resin mixture overnight. After that the cells were completely exchanged in 100% Spurr’s resin twice for 3 h each time, under vacuum. The resin samples were polymerized at 70°C for 48 h. The final block was trimmed, then cut into ultrathin sections (90 nm thickness) using a Leica Ultracut Ultramicrotome (Leica Microsystems, Wetzlar, Germany) with a diamond knife (Diatome, Pennsylvania, USA). Sections were placed onto 200 mesh copper grids and examined using a JEM 1010 instrument (Jeol). Approximately 40 TEM images were taken at 5000× and 10000× magnifications for sample analysis.

### 2.6. STEM-EDS

STEM-EDS was used in the elemental analysis and mapping of the AuNS in the ultrathin sections. STEM-EDS analysis was performed on samples prepared as described above using a Jeol JEM-F200 TEM (FEG, 200 kV). The instrument was equipped with a high-angle annular dark field detector (HAADF, scanning TEM mode, STEM) and an energy-dispersive X-ray spectrometer (EDS).

### 2.7. Cellular morphology

The scanning electron microscope FeSEM SUPRA 40VP (Carl Zeiss, Jena, Germany) with a primary beam energy of 3 kV was used. A 100 µL aliquot of cells in PBS were placed on a glass cover slip (ProSciTech, Kirwan, Australia) in duplicate. The glass cover slips were then washed with nanopure H_2_O (resistivity of 18.2 MW cm^−1^) and dried with 99.99% purity nitro­gen gas. The PC 12 cells exposed to 10 min of SS THz radiation were fixed in a cocktail of 2.0% para­formaldehyde and 2.5% glutaraldehyde for 30 min. The cells were then dehydrated by passing through a graded ethanol series (20%, 40%, 60%, 80% and 100%) for 15 min. Before imaging, the fixed cells were subjected to gold sputtering (7 nm thick) using a NeoCoater MP-19020NCTR (Jeol, Tokyo, Japan). The same procedure was applied to non-treated PC 12 cells.

### 2.8. Cell viability

The viability of PC 12 cells was determined using the LIVE/DEAD Viability/Cytotoxicity Kit (Invitrogen) according to the manufacturer protocol. The viability of the SS THz irradiated cells and the controls was monitored immediately after the treatment and confirmed through three technical replicates. CLSM was used in assessing the number of viable cells; approximately ten fields of view were analysed per sample type and the number of cells per mm^2^ were expressed.

### 2.9. Statistical analysis

Statistical data processing was conducted using the *Statistical Package for the Social Sciences*, *SPSS 24.0* (SPSS, Chicago, IL, USA). Statistically significant differences (*p* < 0.05) among the various groups were calculated by independent groups t-test. The independent variable was the different treatment conditions (Control, SS THz). Results were gathered from three repeats and two technical repeats and averaged, where the average was used as a dependent variable.

## 3. Results and discussion

PC 12 cells were exposed to 10 min of SS THz radiation at the BEP as illustrated in Fig. 1 ▸. The average temperature in the presence of THz radiation was recorded to be 24.57 ± 0.12°C, which was considered to negligible given that the increase was insignificant, only in the range of 0.12°C. When exposing water-based samples, such as biological tissues, or water-based solutions containing samples, the absorption coefficient of water (α_w_) needs to be considered, as this significantly alters both the intensity and the frequency profile at a given sample depth. α_w_ increases from approximately 150 cm^−2^ at 0.5 THz to 3000 cm^−2^ (Segelstein, 1981 ▸). The consequence is that, for all practical purposes, all frequencies are absorbed within the first 100 µm, with the higher THz frequencies absorbed closer to the surface of the sample when compared with the lower frequencies. This results in significant differences in the exposure profile both in terms of sample depth and frequency. The changes in the THz radiation absorption pattern are presented in Fig. 1 ▸(*d*). In order to present a uniform THz radiation exposure profile to all cells, a monolayer of PC 12 cells was created on a thin polyethyl­ene film using an O-ring of radius 2.5 mm (giving a surface area of approximately 17.2 mm^2^) and exposed to the beam. Since the PC 12 cells are approximately 10 µm in diameter, the cells would receive a reasonably uniform exposure profile equivalent to the sample depth of 5 µm. An exposure of 10 min with a 1.0 W m^−2^ incident beam over an area of 17.2 mm^2^ results in a total energy input of approximately 10.3 mJ.

**Figure 2:**
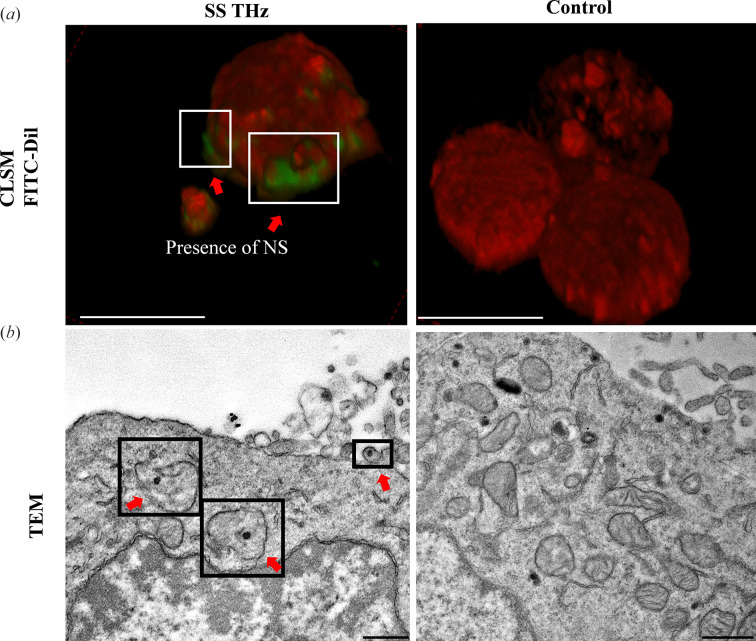
SS THz induced NS uptake by PC 12 cells confirmed by CLSM and TEM. (*a*) Confocal micrographs displaying FITC NS uptake. The presence of NS near the membrane (white insets) is confirmed by the presence of the green signal (red arrows). Scale bars: 10 µm. (*b*) TEM micrographs displaying AuSi NS uptake (red arrows). NS appear to be localized inside the cytoplasm and closer to the cell membrane. Scale bars: 0.5 µm.

Cell membrane permeability in PC 12 cells as a result of SS THz radiation was confirmed using fluorescence imaging and electron microscopy as illustrated in Fig. 2 ▸. Internalization of FITC NS was seen using confocal microscopy where the green signal was observed bound to the outer membrane (white insets) [Fig. 2 ▸(*a*)]; similar results were obtained before in response to SS THz radiation (Perera *et al.*, 2019*a* ▸) whereas the unexposed control sample did not indicate the presence of NS. The high-resolution TEM technique was used as an alternative technique, where ultrathin section of cells revealed the presence of NS in vacuoles [Fig. 2 ▸(*b*), black insets, red arrows] inside the cell cytoplasm. By contrast, the untreated control sample did not exhibit NS internalization.

**Figure 3:**
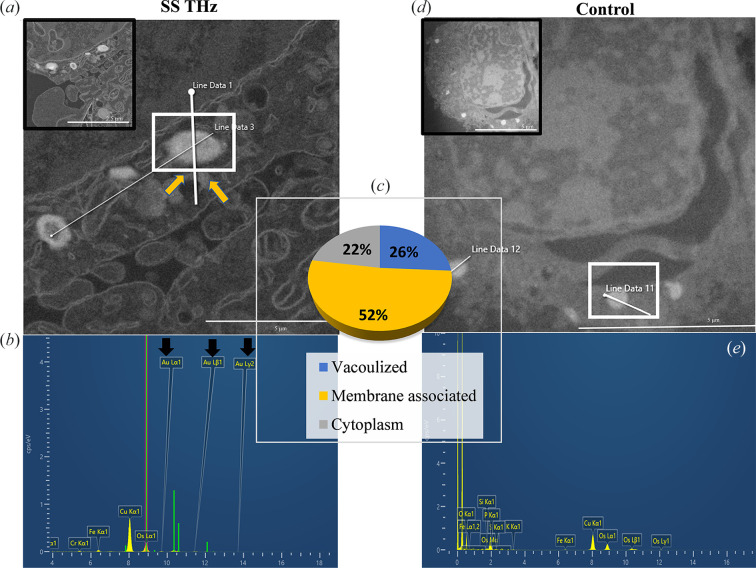
Localization of silica core-shell Au NS in PC 12 following exposure to SS THz radiation. (*a*) STEM micrographs illustrating SS THz irradiated PC 12 cells. NS are seen internalized in vacuoles (yellow arrows) inside the cytoplasm. (*b*) EDS spectra representative of the line data 1 in the electron microscopy image (*a*) confirming the presence of elemental Au (black arrows), thus confirming the presence of AuSi NS localized inside vacuoles in the cellular cytoplasm. (*c*) Quantification of the localization of AuSi NS. The majority of the NS are present in the cytoplasm and are sequestered in vacuoles. (*d*) STEM micrograph of the unexposed PC 12 cell sample and (*e*) representative EDS spectra. No peaks were detected for Au, line data 11, confirming the absence of Au inside the vacuoles that were observed inside the control cells.

Following confirmation of the Si NS cell–particle interactions, localization of the AuSi NS was further explored using STEM-EDS (Fig. 3 ▸). Vacuoles in PC 12 cells are visible in the STEM-EDS micrograph [Fig. 3 ▸(*a*), white insets, yellow arrows], and the line data spanning the entire vacuole reveal the presence of elemental Au [Fig. 3 ▸(*b*), black arrows] in the corresponding spectra. Quantification of the NS localization [Fig. 3 ▸(*c*)] revealed that most of the NS were membrane bound (52%) while 22% were in the cytoplasm and the rest were sequestered in vacuoles (26%); similarly, following exposure to HF EME of 18 GHz, AuSi NS were found to be mainly associated with the membrane after being vacuolized (Perera *et al.*, 2018 ▸; Tharushi Perera *et al.*, 2022 ▸).

In contrast, the non-exposed PC 12 cells [Fig. 3 ▸(*d*)] were analysed using line data spanning the vacuoles inside the cell, where the corresponding spectra [Fig. 3 ▸(*e*)] did not display any elemental peaks for Au; thus, confirming the absence of AuSi NS in the interior of the cells, which was also confirmed previously (Perera *et al.*, 2019a ▸). The exposure time causing this effect with 18 GHz was only 30 s (which was repeated thrice, with cooling periods of 2 min) whereas the THz exposure lasted for 10 min. This prolonged exposure may drive the endocytic process of the PC 12 cells and may have led to more NS coming into contact with the irradiated cells being vacuolized, which would explain why the SS THz exposed cells exhibited a higher number of NS vacuolizations in comparison with the 18 GHz exposed PC 12 cells. The current results suggest that the NS reside mainly in the cytoplasm where the membrane bounded endosomes have evolved into lysosomes (Chu *et al.*, 2014 ▸). As reported previously in the literature, spherical NPs (as used in the current study) remain stable in endosomes following endocytosis and evolve with the maturation of endosomes (Chu *et al.*, 2014 ▸), and can be easily excreted following exocytosis (Yanes *et al.*, 2013 ▸).

**Figure 4:**
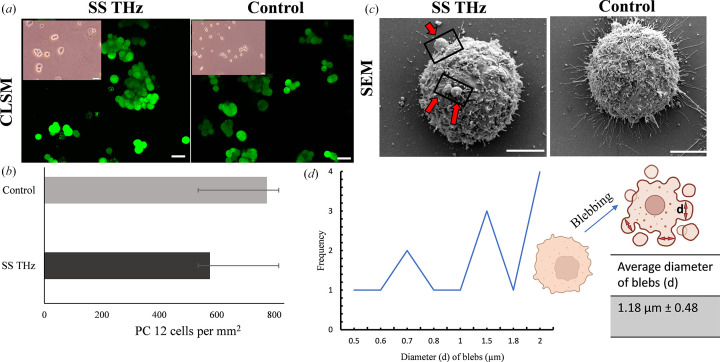
PC 12 cells viability and morphology following exposure to SS THz radiation. (*a*) Optical micrographs (insets) of PC 12 cells exposed to SS THz radiation and the control. Scale bars: 5 µm. CLSM micrographs of viable PC 12 cells are shown in green – the cells were stained and imaged after exposure to SS THz; the untreated control is seen on the right. (*b*) Quantification of viable PC 12 cells. No statistically significant differences were observed among the two experimental groups (*p* = 0.28). (*c*) SEM micrographs depicting PC 12 cell morphology. Blebs were present in the outer membrane of the PC 12 cells in response to SS THz radiation, highlighted in the black insets by red arrows. (*d*) Quantification of the diameter *d* as shown in the insets (normal cell followed by blebbing). The average size of the blebs was 1.08 ± 0.49 µm.

The viability of PC 12 cells has been previously shown in response to ionizing radiation at a fixed frequency of 18 GHz (Perera *et al.*, 2019a ▸); the viability of the cells following exposure to SS THz radiation was confirmed in the current study [Figs. 4 ▸(*a*) and 4(*b*)].

Following exposure to SS THz radiation, viability of the PC 12 cells remained unaffected in comparison with the non-exposed cells; quantification [Fig. 4 ▸(*b*)] of the viable PC 12 cells revealed that the number of viable cells following exposure was reduced in number, but the findings were not statistically significant (*p =* 0.28). Another interesting finding, which is consistent with what has been shown previously (Perera *et al.*, 2019a ▸), is the blebbing of PC 12 cells as a result of SS THz exposure [Fig. 4 ▸(*c*); black insets and red arrows]. Examination of the blebs indicated that the diameter *d* of the protrusions of the exterior membrane ranges from 0.4 to 2.0 µm with an average diameter of 1.18 ± 0.48 µm.

The findings of the current study confirmed induced permeability in PC 12 cells following exposure to SS THz radiation without compromising cellular viability and morphology, which is in agreement with our previous reports using mammalian cells (Perera *et al.*, 2018 ▸, 2019*a* ▸) and prokaryotic cells (Nguyen *et al.*, 2015 ▸, 2016 ▸; Shamis *et al.*, 2011 ▸). Few other cell types and biological systems including *in vivo* models have been studied (Jhawar *et al.*, 2022 ▸); one study demonstrated that exposure to 0.72 THz for 60 min resulted in changes of adhesion and axon structure in lymnaea stagnalis neurons (Perera *et al.*, 2019*a* ▸). In another study, human dermal fibroblasts exposed to continuous THz radiation from an optically pumped molecular gas THz laser source (2.52 THz, 227 mW cm^−2^) for up to 80 min were shown to be 90% viable (Perera *et al.*, 2019 ▸ *b*; Nguyen *et al.*, 2017 ▸; Tharushi Perera *et al.*, 2021 ▸). When exposed to the narrow-band THz radiation (2.3 THz, 1.4 W cm^−2^), human embryonic stem cells showed no structural chromosomal aberrations or difference in cell morphology when compared with the untreated control, and only some minor up-regulations of mitochondria-related genes were reported (Nguyen *et al.*, 2015 ▸).

Poration of the cell membrane plays an important role in the intracellular delivery of molecules of various sizes (Fan *et al.*, 2015 ▸), and has been used in breaching the cell membrane by alternative techniques including electroporation, sonoporation and mechanical stress (Fan *et al.*, 2015 ▸; Bolhassani *et al.*, 2014 ▸; Tomizawa, 2013 ▸). Electroporation works by applying short external pulsed electric fields to create pores in the membrane (Kotnik *et al.*, 2015 ▸); sonoporation, on the other hand, uses sound waves instead of electric pulses (Tomizawa, 2013 ▸). SS THz radiation induces membrane permeability, not by creating pores in the membrane but rather because its non-ionizing radiation seems to have an effect on the fluidity of the cell membrane that leads to increased permeability, which is an independent mechanism (Tharushi Perera *et al.*, 2022 ▸). Drawbacks of the current (alternative) techniques include having a negative effect on normal cellular functions and cell viability, and in the case of electroporation it can cause permanent permeabilization that leads to cell death and strong electrochemical reactions (Zu *et al.*, 2014 ▸). THz and GHz radiation, on the other hand, facilitate foreign material into the cells without undermining cellular viability, intra-cellular physiology, morphology or neuronal differentiation [based on results using PC 12 cells (Perera *et al.*, 2019*a* ▸)]. Another disadvantage of using these techniques is that there is no control over the area which the technique is applied to (Zu *et al.*, 2014 ▸; Towhidi *et al.*, 2016 ▸), whereas non-ionizing EME including SS THz and GHz can be localized to a specific location, with the entire sample or the monolayer receiving an equal amount of radiation and thus uniform energy absorption. Thus, EME in the THz range has an immense potential in the field of drug delivery and targeted therapy as a safe and effective technique of membrane permeabilization.

The mechanism responsible for non-ionizing radiation-induced membrane permeability remains unclear due to the lack of the relevant data on non-ionizing studies. It may be speculated that cell blebbing or membrane protrusions can be used as an indication of how the membrane responds to HF EME as a result of a drop in intra-cellular pressure (Charras, 2008 ▸; Norman *et al.*, 2010 ▸). Blebs or cellular protrusions may occur and disappear, and are the result of actomyosin contractions of the cortex which leads to transient detachment of the cell membrane from the actin cortex (Charras, 2008 ▸; Norman *et al.*, 2010 ▸). In the growth of a bleb, lipids will then flow into the bleb through its neck and the actin cortex is reconstituted (Charras, 2008 ▸; Norman *et al.*, 2010 ▸).

HF EME has been shown to influence peptides molecules’ dipole moment (Todorova *et al.*, 2016 ▸). Simulation studies have shown that high-strength electromagnetic radiation in the range >0.04 V nm^−1^ led to structural changes in peptides (Todorova *et al.*, 2016 ▸) given that upon absorption of EME a molecule undergoes three possible transitions: rotational, vibrational or electronic (Tuieng *et al.*, 2021 ▸). EME will also be absorbed by water dipoles (Tuieng *et al.*, 2021 ▸) and the increased vibration of the charged molecules may in turn have an effect on other cellular components that leads to changes in cell membrane fluidity (Shamis *et al.*, 2012*b* ▸; Tuieng *et al.*, 2021 ▸). Phospho­lipids may also play a role in the process of endocytosis (Bohdanowicz & Grinstein, 2013 ▸), yet the mechanism as to how EME induces cell membrane permeability is yet to be explored.

## 4. Conclusions

The current findings revealed that the majority of AuSi NS were present in the cytoplasm of SS THz exposed cells as single NS or clusters, with the rest accumulated in vacuoles. Since the formation of vesicles containing NS is evident in the irradiated samples, it can be stated that SS THz induced translocation could trigger endocytosis leading to NS uptake and internalization in the form of membrane-bound vesicles which later evolve with the maturation of endosomes. Given the current study, SS THz radiation facilitates the delivery of NS in the range 20–50 nm making it a safe, effective technique for cell therapy and drug delivery.
